# The Anti-Migratory Effects of FKBPL and Its Peptide Derivative, AD-01: Regulation of CD44 and the Cytoskeletal Pathway

**DOI:** 10.1371/journal.pone.0055075

**Published:** 2013-02-15

**Authors:** Anita Yakkundi, Lynn McCallum, Anthony O’Kane, Hayder Dyer, Jenny Worthington, Hayley D. McKeen, Lana McClements, Christopher Elliott, Helen O. McCarthy, David G. Hirst, Tracy Robson

**Affiliations:** 1 School of Pharmacy, Queen’s University Belfast, Northern Ireland, United Kingdom; 2 Institute for Global Food Security, School of Biological Sciences, Queen’s University Belfast, Northern Ireland, United Kingdom; 3 Biomedical Sciences, University of Ulster, Coleraine, Northern Ireland, United Kingdom; University Hospital of Modena and Reggio Emilia, Italy

## Abstract

FK506 binding protein-like (FKBPL) and its peptide derivatives exert potent anti-angiogenic activity *in vitro* and *in vivo* and control tumour growth in xenograft models, when administered exogenously. However, the role of endogenous FKBPL in angiogenesis is not well characterised. Here we investigated the molecular effects of the endogenous protein and its peptide derivative, AD-01, leading to their anti-migratory activity. Inhibition of secreted FKBPL using a blocking antibody or siRNA-mediated knockdown of FKBPL accelerated the migration of human microvascular endothelial cells (HMEC-1). Furthermore, MDA-MB-231 tumour cells stably overexpressing FKBPL inhibited tumour vascular development *in vivo* suggesting that FKBPL secreted from tumour cells could inhibit angiogenesis. Whilst FKBPL and AD-01 target CD44, the nature of this interaction is not known and here we have further interrogated this aspect. We have demonstrated that FKBPL and AD-01 bind to the CD44 receptor and inhibit tumour cell migration in a CD44 dependant manner; CD44 knockdown abrogated AD-01 binding as well as its anti-migratory activity. Interestingly, FKBPL overexpression and knockdown or treatment with AD-01, regulated CD44 expression, suggesting a co-regulatory pathway for these two proteins. Downstream of CD44, alterations in the actin cytoskeleton, indicated by intense cortical actin staining and a lack of cell spreading and communication were observed following treatment with AD-01, explaining the anti-migratory phenotype. Concomitantly, AD-01 inhibited Rac-1 activity, up-regulated RhoA and the actin binding proteins, profilin and vinculin. Thus the anti-angiogenic protein, FKBPL, and AD-01, offer a promising and alternative approach for targeting both CD44 positive tumours and vasculature networks.

## Introduction

Angiogenesis is the formation of new blood vessels from pre-existing vessels and its regulation is an essential component of various developmental and wound healing pathways [1]; loss of this regulation forms the basis of many pathological conditions [2]. Anti-angiogenic agents in clinical use target the VEGF pathway; Macugen and Lucentis [3] for the treatment of macular degeneration and Avastin [4], sunitinib [5] and sorafenib [6] for targeting tumour growth. However, these VEGF-targeted anti-cancer agents elicit modest response rates, generate resistance and stimulate tumour metastasis in some instances [7]. Due to the observed toxic effects [8] and limited efficacy, the FDA has recently voted for the withdrawal of Avastin for metastatic breast cancer treatment [9], [10]. This highlights the demand for better anti-angiogenic intervention strategies. We have recently reported the development of novel peptides based on the anti-angiogenic domain of the FKBPL protein. These have potent anti-angiogenic activity but appear to target a CD44-dependent pathway, differentiating them from the VEGF-targeting agents [11].

FKBPL belongs to the FK506 family of immunophilins [12], [13], with homology spanning the tetra trico peptide repeat (TPR) domain which mediates its interaction with HSP90 [14]. FKBPL plays a critical role in regulating steroid receptor complexes; glucocorticoid [15], androgen [16] and estrogen receptors [17]. Its regulation of estrogen signalling supported a role for FKBPL as a prognostic and/or predictive biomarker of response to endocrine therapy in breast cancer patients [18]. Our most recent data also support an extracellular role for this protein in the regulation of angiogenesis [11]. The active anti-angiogenic domain was localised to a region distinct from the TPR domain and a therapeutic peptide, AD-01, spanning this domain was developed. A preclinical candidate based on AD-01 has successfully completed toxicology studies with a view to initiating Phase I clinical trials for solid tumours.

We have reported the anti-angiogenic activity of exogenously administered FKBPL/AD-01 and their dependency on CD44 [11]. Here, we have further characterised the activity of the endogenous protein as well as demonstrating an interaction between FKBPL and AD-01 with CD44, their effect on the actin-tubulin cytoskeleton and downstream signalling, leading to deregulation of the RhoA-Rac pathway and consequential inhibition of cell migration.

## Materials and Methods

### Cell Culture

HMEC-1 cells were obtained from the Centre of Disease Control (Atlanta, USA); PC3 and MDA-MB-231 from the American Type Culture Collection (Manassas, VA, USA). FKBPL overexpressing MDA-MB-231 cells were derived from MDA-MB-231 cells as described earlier [11]. All cell lines were authenticated by short tandem repeat (STR) profiling carried out by the suppliers and routinely tested for *Mycoplasma*. All cell lines were cultivated at 37°C in a humidified atmosphere of 5% CO_2/_95% O_2_ in appropriate medium supplemented with 10% fetal calf serum (FCS) (PAA, UK); HMEC-1 cells in MCDB-131, (Invitrogen, UK), supplemented with epidermal growth factor (EGF, 10 ng/ml) (Roche, UK) and L-glutamine (10 mM) (Invitrogen, UK); MDA-MB-231 cells in DMEM (Invitrogen, UK) and MDA-MB-231 FKBPL overexpressing cells were additionally supplemented with G418 (Sigma, UK); PC3 cells in RPMI 1640 (Invitrogen, UK).

### Peptide Synthesis

AD-01 (NH_2_-QIRQQPRDPPTETLELEVSPDPAS-OH) and scrambled AD-01 were synthesized and supplied by Almac Group [11]. Purified AD-01 had >95% purity and an observed mass of 2704.0 kDa.

### Cell Migration Assay

The *in vitro* migration assay is a modified version of the method described by Ashton *et al* [19]. HMEC-1 or MDA-MB-231 cells were plated in chamber slides (Lab-Tek®, UK) and the confluent monolayer was wounded using a sterile pipette tip and cells treated with drug/antibody. The HMEC-1 monolayers were incubated up to 12 h for the time course experiment and MDA-MB-231 monolayers for 18 h (∼50% wound closure) after treatment. The extent of “wound” closure was assessed blindly as previously described [11]; 3 slides were processed in each treatment group and the entire experiment repeated in triplicate. Data are expressed as % wound size normalised to time zero (T_0_) control or as % inhibition compared to a time matched control.

### Subcellular Fractionation

Cytosol, membrane, cytoskeletal and nuclear fractions from HMEC-1 and MDA-MB-231 monolayers were harvested using the ProteoExtract® Subcellular Proteome Extraction Kit (Calbiochem, UK, Cat. no.539791). Specificity of the fractions was determined using specific antibodies; Vimentin, Calpain and Histone H1 diluted at 1∶1000.

### Western Blot Analysis

Log phase HMEC-1 or MDA-MB-231 cells were treated with AD-01 at the stated dose and time or HA at 0.1 mg/ml (234 kDa, Life core Biomedicals, USA) and cell lysates harvested using RIPA (Radio-Immuno Precipitation Assay) buffer supplemented with protease and phosphatase inhibitor cocktails (Roche, UK). Cell lysates were reduced in Laemmli buffer (Sigma, UK) and subjected to western blotting as reported earlier [11]. Blots were probed with specific primary antibody and appropriate HRP-linked secondary IgG (GE Healthcare, UK) at 1∶10000, followed by detection with Chemiluminescent Substrate (Millipore, UK).

### Antibodies

CD44H (R&D Systems, cat: BBA10); FKBPL (Proteintech, USA cat: 10060-1-AP); vinculin, profilin, cofilin, p-cofilin (Cell signalling, cat: 4650, 3237, 3318 and 3313, respectively); RhoA (BD Transduction, cat: 610848); anti-GAPDH and Actin (Sigma, UK, cat: G9545 and A4700 respectively); Rac-1 (Millipore, MA cat: 05–389). Histone H1 was obtained from Santa Cruz Biotechnology. Polyclonal anti-AD-01 antibody was supplied by Almac Discovery. All antibodies were used at 1∶1000 unless otherwise stated.

### Rac Assay

This was carried out as described in our earlier paper [11]. Briefly, HMEC-1 monolayers were serum starved overnight, pre-treated with AD-01 (10^−9^ M) for 10 min/60 min, prior to stimulation with fMLP (1 µM, Sigma, UK) for 30 sec at 37°C and lysates subjected to Rac GTPase assay (Millipore, UK).

### Flow Cytometry

MDA-MB-231 cells were harvested using the non-enzymatic cell detachment buffer (Invitrogen, UK) and suspended at 2×10^5^ cells/100 µl, in phenol red free-DMEM with 1% BSA (bovine serum albumin (Sigma,UK), flow buffer). Cells were incubated with anti-CD44 antibody at 1–2 µg/ml for 1 h at 4°C on a rotating wheel, washed three times with flow buffer and incubated for a further 30 min at 4°C with Alexa 488-conjugated secondary mouse antibody. The cells were further washed, re-suspended in flow buffer and analysed on a BD LSR II Flow cytometer.

### BIACORE Binding Assay

AD-01 (500 mM) was immobilised on an EDC/NHS activated CM5 chip (GE Healthcare, USA), in the presence of 1 M NaCl and 25 mM borate buffer (pH 8.5). This was followed by end-capping with ethanolamine. The Rmax (∼8000 RU) of anti-AD-01 antibody (50 µg/ml) binding was assessed over a 10 min injection period at 5 µl/min. In order to detect displacement of the antibody/AD-01 interaction by CD44, a significantly lower concentration of antibody was necessary (0.01 µg/ml). Binding of the diluted antibody was inhibited in the presence of AD-01 (0–1 µM) over a 4–8 min injection period at 10 µl/min. Competition of this inhibition was used as a measure of real-time interaction between AD-01 and CD44. Immunopurified CD44, or cytosol and membrane extracts from MDA-MB-231 cells transfected with CD44-targeted or non-targeted (NT) siRNA were used to assess the interaction; recombinant human EGFR (R & D systems, cat: 344-ER) and a scrambled AD-01 peptide were used as negative controls. Cell extracts were diluted 1∶1 with HBS buffer, mixed with the indicated concentrations of peptide in a 96-well plate format and the complexes further mixed with anti-AD-01 antibody prior to automated injection. Binding analyses were performed on BIACORE Q, using BIACORE Q Control and Evaluation Software. Data transformation was carried out to represent the binding efficiency across 5 independent experiments. The relative binding of anti-AD-01 antibody in the presence of the indicated concentrations of AD-01, was calculated as the percentage of the maximum resonance binding units at 0.001 µM and 0.01 µM AD-01 in each experiment.

### CD44 Immunopurification

CD44 (10 µg) or murine isotype control antibody was crosslinked to agarose beads using CNBr (Pierce, UK) and incubated overnight with cell extracts from MDA-MB-231 (1 mg/ml protein) at 4°C. CD44 was eluted with elution buffer provided with the kit and neutralised with 10 µM NaOH and used immediately for binding assays; frozen immunoprecipitates were inactive.

### Real-time PCR

RNA was extracted using STAT-60™ (AMS Biotechnology, Europe) and reverse transcribed using the high capacity RNA-to-cDNA kit (Applied Biosystems, Foster City, CA, cat 4387406). RQ-PCR was performed using an ABI PRISM 7500 Sequence Detector (Applied Biosystems, UK). Primer probe sets were obtained from Roche Applied Science UK (FKBPL assay: 128226, CD44 assay: 110687 and GAPDH assay: 101128). Reaction mixes (12.5 µl) contained 50 ng cDNA equivalents (or control), 1x Taqman universal PCR master mix, 5 mM MgCl_2,_ 0.2 mM each of dATP, dCTP and dGTP, 0.4 mM dUTP, 0.125 U AmpliTaq Gold, 2 µM primers (forward and reverse) and 200 nM TaqMan probe. Amplifications were performed with an initial 20 sec incubation at 50°C, followed by treatment at 95°C for 10 min. This was followed by 40 cycles of denaturing at 95°C for 15 sec and annealing/extension at 60°C for 1 min. Data were collected and analysed with Sequence Detector v1.6.3 software (Applied Biosystems). Relative quantitative (Q-PCR) data was calculated based on the δδC_T_ method [20].

### siRNA Transfections

MDA-MB-231 cells grown to 50% confluence were transfected with either, 50 or 500 nM FKBPL targeted siRNA (Ambion, UK), 16 nM smart pool CD44 targeted siRNA or equivalent amounts of NT siRNA using Dharmafect 1 (Dharmacon, Chicago, IL). HMEC-1 cells were transfected at 80% confluence using Lipofectin (Invitrogen, UK). Following a 72 h transfection the cells were replated and subjected to either, migration assay, binding assay, or western blotting ± AD-01 (10^−9 ^M) treatment using methods described above.

### Immunoprecipitation

Cell lysates were prepared from HMEC-1 cells using BRIJ lysis buffer (50 mM Tris-HCl, pH 8.0, 62.5 mM EDTA, 0.4% sodium deoxycholate, 1% Brij 58, phosphatase inhibitor cocktail 1 and 2; Sigma P2850 and P5726, respectively). Lysates (1 mg protein) were incubated overnight at 4°C with 1 µg of primary antibody (CD44 or FKBPL) or isotype controls; murine IgG2a (DAKO, UK) and rabbit IgG (DAKO). Isotype control IgGs were used at 1∶1000 concentration of primary antibody. Samples were incubated with Protein A/G (20 µl, Invitrogen, UK) for 2 h at 4°C, washed with BRIJ lysis buffer, re-suspended in Laemmli buffer and subjected to western blotting as described above.

### Confocal Microscopy

MDA-MB-231 or HMEC-1 monolayers were grown to 50–60% confluency in chamber slides (Lab-Tek®, UK), wounded as described above and treated with AD-01 (10^−9 ^M) for 24 h or recombinant FKBPL (rFKBPL) (Fusion Antibody Ltd, UK) (750 ng/ml) for 5 h. Cells were washed with PBS, fixed with 4% paraformaldehyde, permeabilised with 0.1% Triton X-100 (Sigma-Aldrich, UK) and blocked with 2% BSA. This was followed by staining with either TRITC-phalloidin (Millipore, UK), anti-AD-01 (1∶1000) or anti-tubulin (1∶500) antibody for 2 h at room temperature, washed with PBS-T and stained for 2 h at room temperature with a fluorescein isothiocyanate (FITC)/Alexa-488-tagged secondary antibody. Finally, DAPI (1∶1000) or propidium iodide was added for 5 min, slides were mounted with Vectashield mounting medium (Vector laboratories Inc., UK) and analysed on Leica TCS SP5 Microscope (Germany) using 40x or 60x oil immersion objective. Isotype controls included rabbit or mouse IgG (1∶10000).

### Intravital Microscopy

Viewing chambers were inserted under the dorsal skin of female Balb-c SCID mice. Fragments of MDA-MB-231 parental or FKBPL overexpressing tumours were placed on the microvascular bed and covered with a glass microslide. 21 days following tumour implantation, mice were injected i.p. with FITC-dextran and tumour vasculature was imaged using epi-florescence microscopy at 100x and 20x magnification. Intravital images were analyzed by ImageJ software (NIH, USA). Number of vessel branch points or average vessel diameter (µm) were counted from 3D images; n = 5 mice per treatment group, 4 fields of view per tumour and 30 vessels per field. Animal experimentation was carried out under the ethical approval of the Department of Health and Social Service Protection, Northern Ireland taking into consideration the 3 Rs to ensure minimal suffering.

### Statistics and Graphs

Statistical significance was determined either by T–test or one-way or two-way ANOVA and line graphs were generated using Prism Graphpad software.

## Results

### FKBPL is a Natural Anti-angiogenic Protein and is Distributed within Several Intracellular Compartments

**Figure 1:**
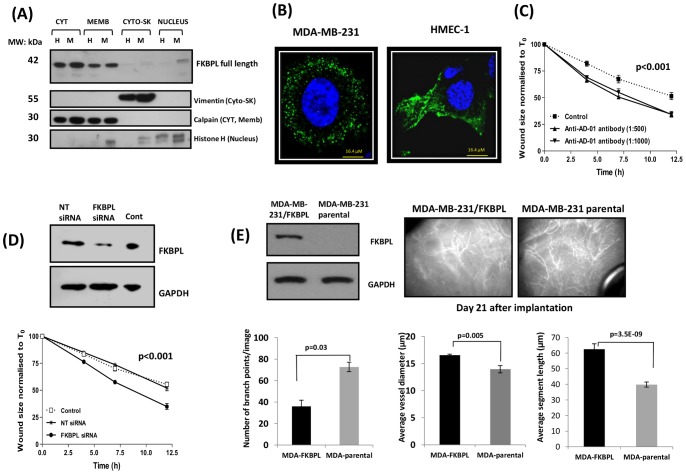
FKBPL is present in various cell compartments and regulates cell migration and tumour vasculature. (**A**) Representative blot demonstrating that FKBPL is present predominantly in the cytosol and membrane compartments of both HMEC-1 (H) and MDA-MB-231 (M) cells and in the nuclear fraction of MDA-MB-231 cells**.** Protein extracts from each subcellular compartment probed with specific compartmental markers, vimentin, calpain and histone-H1 were used as loading controls. (**B**) Representative confocal images (60x) of MDA-MB-231 and HMEC-1 cells fixed, permeabilised and stained with DAPI (blue) and with anti-AD-01 primary antibody and Alexa-488 tagged secondary antibody demonstrating vesicular staining for FKBPL (green); n = 3. (**C**) Anti-AD-01 antibody targets the active domain of FKBPL and accelerates HMEC-1 cell migration in comparison to cells treated with an isotype control. Data points show means ± SEM; n = 3 (**D**) FKBPL knockdown with siRNA accelerated migration of HMEC-1 cells in comparison to un-transfected and NT-siRNA-transfected cells. Data points show means ± SEM; n = 3. Cell migration was assessed using scratch wound assay. Wound size is normalised to that of T_0_. p-value was determined using two-way ANOVA. (**E**) Intravital microscopy images (20x) representing disruption of tumour vasculature *in vivo* in FKBPL-overexpressing MDA-MB-231 xenografts in comparison to those derived from parental MDA-MB-231 cells. Tumours (21 days) were imaged using Epi-fluoresence microscopy following injection of mice with FITC-Dextran. Quantification of vessel dynamics was carried out on 3D images using ImageJ software. n = 5 mice per treatment group (p-value was determined using two-tailed T –test).

We have previously shown that FKBPL is secreted from HMEC-1 and lung epithelial cells, L132, but not from the tumour cell line, MDA-MB-231 [11]. To further characterize endogenous FKBPL in terms of its cellular location and function, we carried out western blot analysis on sub-cellular fractions of HMEC-1 and MDA-MB-231 cells to gain insight into its localisation. Fractionated cell extracts identified a wide cellular distribution profile for FKBPL (Fig. 1A). It was present predominantly in the cytoplasm and membrane of HMEC-1 and MDA-MB-231 cells at the expected molecular weight (∼42 kDa). Low levels were also detected in the nuclear fraction of MDA-MB-231 cells. The specificity of sub-cellular fractions was demonstrated by immunoblotting with specific compartmental markers which also acted as loading controls for the samples used; vimentin for cytoskeletal fractions, histone H1 for nuclear fractions and calpain for both cytoplasmic and membrane fractions in this instance (Fig. 1A). Furthermore, examination of FKBPL distribution by confocal microscopy provided evidence of a punctate staining pattern possibly indicating its presence in vesicular structures within the cytoplasm (Fig. 1B) consistent with that observed for other secretory proteins [21], [22]. Acceleration of wound closure was observed when HMEC-1 cells were treated exogenously with an antibody (Fig. 1C) raised against the active domain of FKBPL (anti-AD-01 antibody), suggesting that this antibody possibly binds to secreted FKBPL to inhibit its anti-migratory activity. The role of endogenous FKBPL in cell migration was further evaluated using FKBPL targeted siRNA. In HMEC-1 cells, knockdown of FKBPL expression accelerated 50% wound closure by 4 h in comparison to untransfected or NT siRNA transfected cells (Fig. 1D). Conversely, in an *in vivo* experiment, xenografts of MDA-MB-231 cells stably overexpressing FKBPL were imaged 21 days after implantation, following injection with FITC-dextran. Intravital microscopy demonstrated impaired vascular development in FKBPL overexpressing tumours compared to MDA-MB-231 controls (Fig. 1E). FKBPL overexpressing xenografts had increased vessel diameter and segment length whilst the number of branch points was significantly reduced, indicative of reduced vessel density which correlated with reduced tumour growth (data not shown) in comparison to parental xenografts. These results provide further evidence of the anti-angiogenic effects of tumour-secreted FKBPL exerting its activity on murine vascular development by outside-in signalling, probably via a cell surface receptor.

### FKBPL and AD-01 Interact with CD44

**Figure 2:**
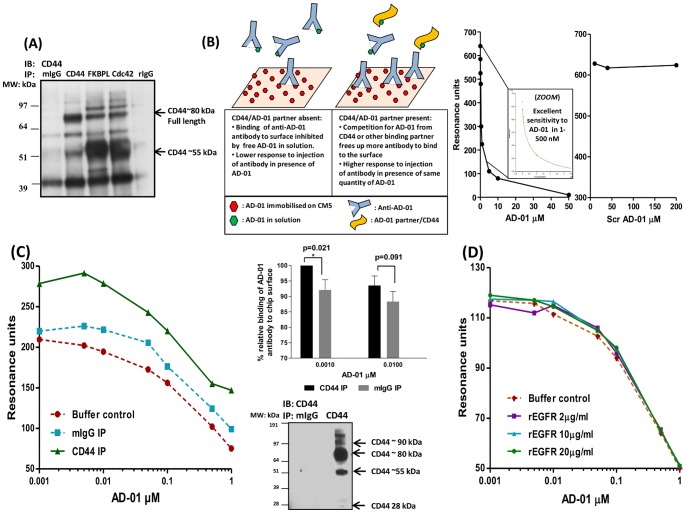
FKBPL and its peptide derivative, AD-01, bind CD44. (**A**) Representative western blot showing FKBPL co-immunoprecipitated with CD44 in HMEC-1 cells; immuno-blotted with anti-CD44 antibody; n = 3; Cdc42 was used as a positive control for the CD44 interaction and rabbit and murine IgGs were used as negative controls. (**B**) Schematic diagram of the Biacore assay using AD-01 immobilised on CM5 chip surface. Binding of anti-AD-01 antibody to AD-01-CM5 surface was inhibited by AD-01 in solution in a dose dependent manner, with excellent sensitivity in lower concentration range of peptide; 1–500 nM. Scrambled AD-01, used as a negative control, did not demonstrate any binding to anti-AD-01 up to 200 µM. Competition of the anti-AD-01 antibody interaction with its cellular partner/s results in increased binding of anti-AD-01 antibody on chip surface. (**C**) Representative graph demonstrating that AD-01 specifically binds to CD44 immunoprecipitated from MDA-MB-231 cells using the assay described. CD44 was immuno-purified from cell lysate and analysed using Biacore Q. Isotype control mIgG antibody was used as control. Bar charts show the relative binding of anti-AD-01 antibody in presence of AD-01, calculated as the percentage of the maximum resonance binding units in the presence of 0.001 and 0.01 µM AD-01. Data points show means ± SEM of 5 independent experiments (p-value was determined by one way ANOVA). (**D**) No competition of anti-AD-01 antibody binding to immobilised AD-01 was obtained in the presence of various concentrations of rEGFR indicating a specificity of AD-01-CD44 interaction.

We have demonstrated previously that rFKBPL and AD-01 mediate inhibition of cell migration in a CD44 dependent manner [11]; however, the interaction with CD44 has not been established. Here, we further investigated the possibility that FKBPL or its peptide derivative, AD-01, could interact with CD44. We used both CD44 and FKBPL antibodies to pull down specific immune complexes in HMEC-1 cells (Fig. 2A). The CD44 antibody immunoprecipitated the ∼55 kDa and the full length 80 kDa CD44 isoforms, consistent with observations from other studies [23], [24]. Interestingly, the FKBPL antibody clearly pulls down the ∼55 kDa and the full length ∼80 kDa isoforms of CD44, suggesting that endogenous CD44 and FKBPL interact either directly or within a complex (Fig. 2A). Cdc42, a known CD44 interacting protein, also pulled down these isoforms of CD44; acting as a positive control. Murine and rabbit IgG were used as negative controls to determine the specificity of the bands pulled by FKBPL and CD44 antibodies and non-specific bands were detected at 39 kDa and 60 kDa in all samples. The other bands shared between FKBPL and Cdc42 are likely to be non-specific. The real-time interaction of AD-01 with CD44 was also assessed in a cell free assay using surface plasmon resonance (BIACORE assay). Unfortunately, commercially available recombinant CD44 was not suitable for this system, probably because the recombinant protein was not fully glycosylated or folded correctly when immobilised to CM5 surface and was therefore unable to bind its natural ligand, hyaluronan (Fig. S1A). A competitive assay system was therefore developed to assess the interaction between AD-01 and CD44 extracted from fresh MDA-MB-231 cell lysates (Fig. 2B). This binding assay was based on the competitive ability of anti-AD-01 antibody to bind to AD-01 on CM5 chip surface in the presence of increasing concentrations of AD-01 in solution. An AD-01 interacting partner such as CD44 should compete with anti-AD-01 antibody binding to AD-01 in solution, allowing the binding of anti-AD-01 antibody to the chip surface which can be detected as an increase in response units (Fig. 2B). AD-01 immobilised to the CM5 chip surface acted as a high affinity ligand for the anti-AD-01 antibody and this binding could be specifically competed by using AD-01 peptide (detectable between 1–100 nM) in solution, in a dose dependent manner (Fig. 2B). Competition was not observed using scrambled AD-01 peptide even at a high concentration (200 µM, Fig. 2B). Using this BIACORE assay, CD44 immunopurified from MDA-MB-231 cellular fractions specifically bound AD-01 in solution resulting in an increased response in comparison to lysates immunopurified with a mIgG isotype or buffer control shown in a representative sensogram chart (Fig. 2C). Due to the variability in absolute sensor units between experiments arising from independently prepared samples and chip surfaces, the data from 5 independent experiments were normalised to the maximum in each group and plotted as bar charts. The combined results showed a significant interaction of immunopurified CD44 in comparison to isotype control, in the presence of 1 nM AD-01 and this was competed out at higher AD-01 concentrations as expected. The inability of recombinant EGFR to affect the binding of anti-AD-01 antibody to the immobilised AD-01 acted as a negative control (Fig. 2D). Thus AD-01 binds specifically to immunopurified CD44 in our binding assay.

### CD44 Knockdown Abrogated AD-01 Binding as well as its Anti-migratory Effect

**Figure 3:**
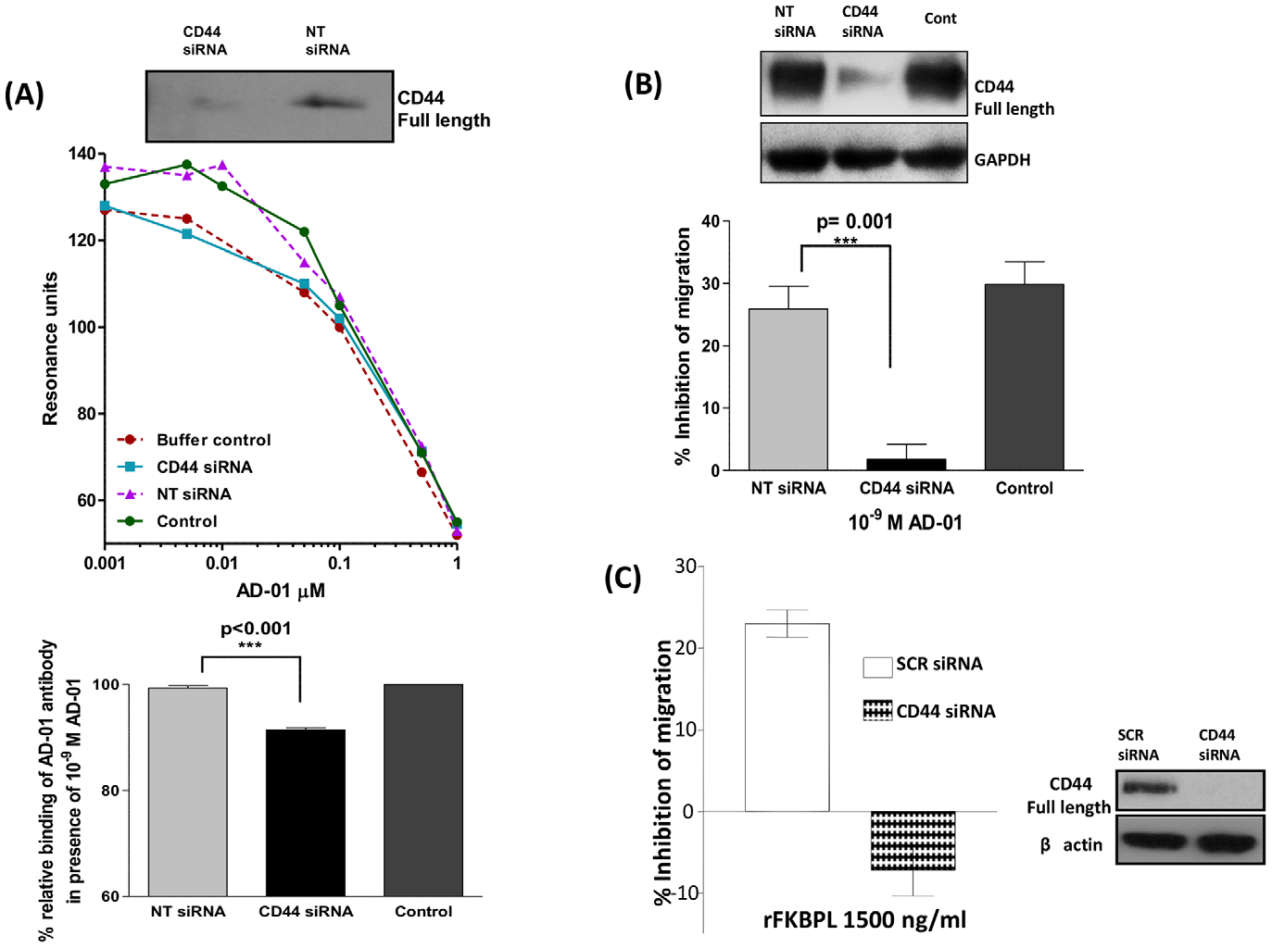
Binding and anti-migratory activity of AD-01 is dependent on CD44. (**A**) Representative graph demonstrating extracts of MDA-MB-231 un-transfected control/NT siRNA transfected cells render increased binding of anti-AD-01 antibody to AD-01 immobilised on CM5 chip surface using the specific binding assay described in Fig. 2. This binding was abrogated in lysates derived from CD44 knockdown cells. Buffer control comprised of HBS buffer with indicated amounts of AD-01. Bar charts demonstrate significant abrogation of this binding in the presence of 10^−9 ^M AD-01 in lysates from cells transfected with CD44 siRNA. Data points show means ± SEM; n = 4. (**B**) Treatment with AD-01 at 10^−9^ M results in inhibition of migration in un-transfected and NT siRNA transfected MDA-MB-231 cells. This inhibition of migration was abrogated upon CD44 knockdown using targeted siRNA. Data points show means ± SEM; n = 5. (p-value was determined using one-way ANOVA). (**C**) Anti-migratory effect of rFKBPL 1500 ng/ml was abrogated in PC3 cells transfected with CD44 targeted siRNA in comparison to those transfected with scrambled siRNA; n = 3.

To determine if AD-01 binding was truly CD44 dependent, MDA-MB-231 extracts from cells transfected with CD44 targeted siRNA, NT siRNA and control cells were subjected to the BIACORE assay. Crude extracts from control or NT siRNA demonstrated increased binding in comparison to the buffer control. However, this binding was abrogated in cell extracts derived from MDA-MB-231 cells transfected with CD44 targeted siRNA (Fig. 3A). Furthermore, CD44 knockdown corresponded phenotypically, resulting in abrogation of the AD-01 mediated inhibition of cell migration in the same cell line, whilst AD-01 was effective at inhibiting migration of control cells and cells transfected with NT siRNA (Fig. 3B). The anti-migratory effect of rFKBPL also showed a dependency on CD44 expression in PC3 cells transfected with CD44 targeted siRNA (Fig. 3C), indicating that FKBPL/AD-01 can mediate anti-migratory effects in tumour cells by targeting CD44.

### FKBPL and AD-01 Regulate Expression of CD44

**Figure 4:**
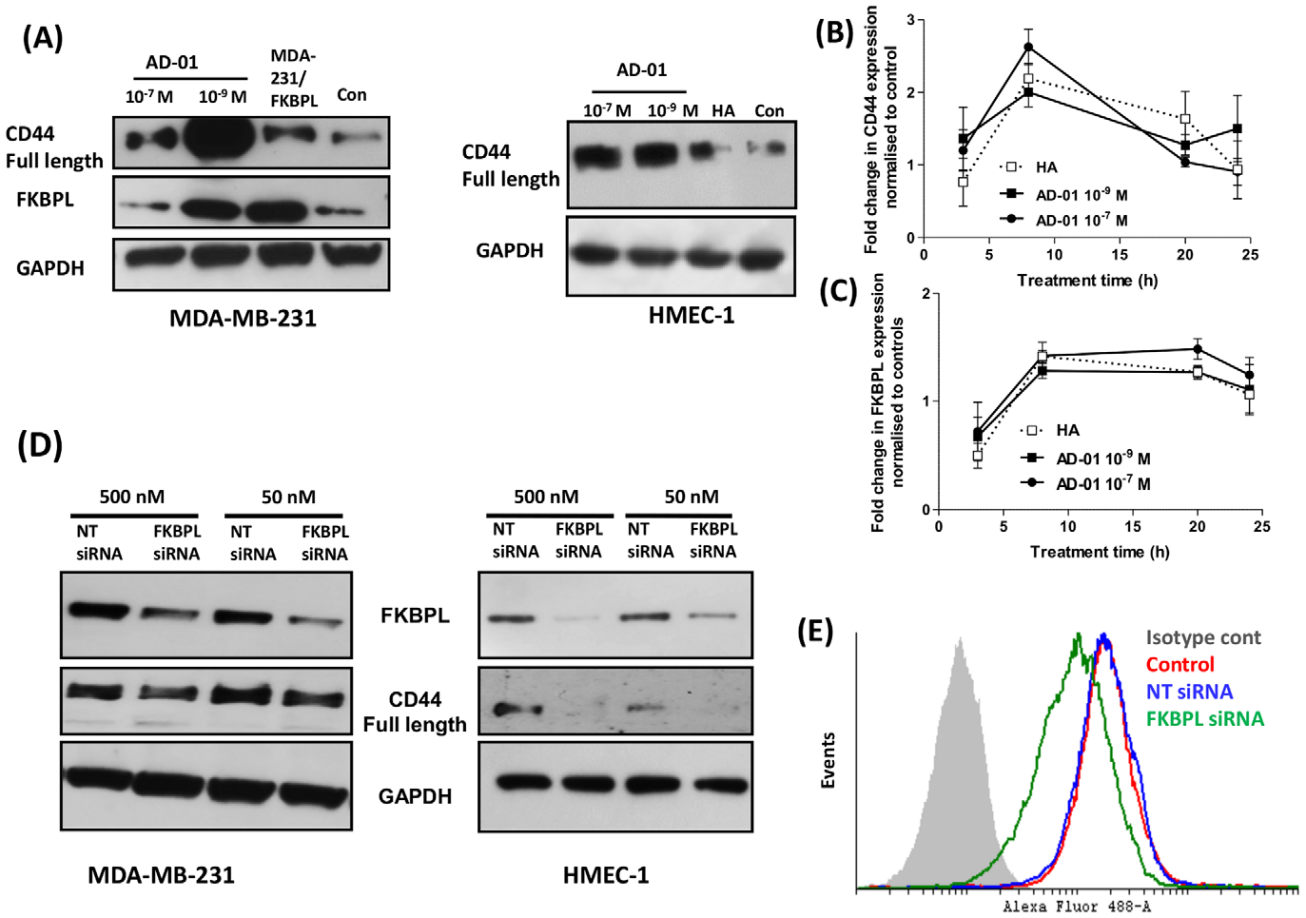
AD-01 and FKBPL regulate CD44 expression. (**A**) Treatment with AD-01 up-regulated CD44 expression. Representative blots of cell lysates from MDA-MB-231/HMEC-1 treated with AD-01 or HA (0.1 mg/ml) for 24 h and MDA-MB-231/FKBPL overexpressing cells, probed with anti-CD44, FKBPL or GAPDH antibodies (n = 3). Transcriptional regulation of CD44 (**B**) and FKBPL (**C**) upon treatment with AD-01/HA was assessed using quantitative RT-PCR. Data points show mean fold change ± SEM in FKBPL or CD44 expression in cDNA prepared from cells treated with AD-01 in comparison to control untreated cells; n = 3. (**D**) Representative western blots demonstrating dose dependent down-regulation of FKBPL upon transfection with FKBPL targeted siRNA for 72 h, resulting in a corresponding down-regulation of CD44 protein; n = 3. (**E**) Flow cytometric analysis of cell surface expression of CD44 in MDA-MB-231 cells transfected with FKBPL siRNA in comparison to NT siRNA and stained with anti-CD44 and Alexa-488 conjugated secondary antibody; n = 3.

To further investigate the molecular effects of AD-01 we considered the potential for AD-01 to alter CD44 expression. Treatment of MDA-MB-231 and HMEC-1 cells with AD-01 (10^−9^ and 10^−7^ M) resulted in an increase in CD44 protein levels; similar increases in CD44 were seen in MDA-MB-231 cells stably overexpressing FKBPL (Fig. 4A). Interestingly, AD-01 (10^−9^ M) also up-regulated FKBPL levels in MDA-MB-231 cells. These effects were also observed at the transcriptional levels; in HMEC-1 cells, an up-regulation of CD44 at the mRNA level by qRT-PCR was observed 8 h after treatment with AD-01 (Fig. 4B); the CD44 ligand, HA, also mediated an increase in CD44 mRNA levels but the up-regulation at the protein level was marginal in comparison to the effects mediated by AD-01. Furthermore, AD-01 and HA also mediated a 1.5 fold increase in FKBPL mRNA levels in HMEC-1 cells (Fig. 4C). Further, we investigated the effect of endogenous FKBPL on CD44 expression. A dose dependent knockdown of FKBPL was observed using 50 and 500 nM FKBPL targeted siRNA. Concomitant with the level of FKBPL knock-down there was a reduction in the expression of CD44 protein in the MDA-MB-231 and HMEC-1 cells (Fig. 4D). Finally, FKBPL silencing also decreased cell surface CD44 levels in comparison to control cells transfected with NT siRNA when MDA-MB-231 cells were assessed by flow cytometry (Fig. 4E). Thus endogenous FKBPL as well as AD-01 regulate the expression of CD44.

### AD-01 Disrupts the Actin Cytoskeleton and Creates an Imbalance in the Rho-Rac Pathway

**Figure 5:**
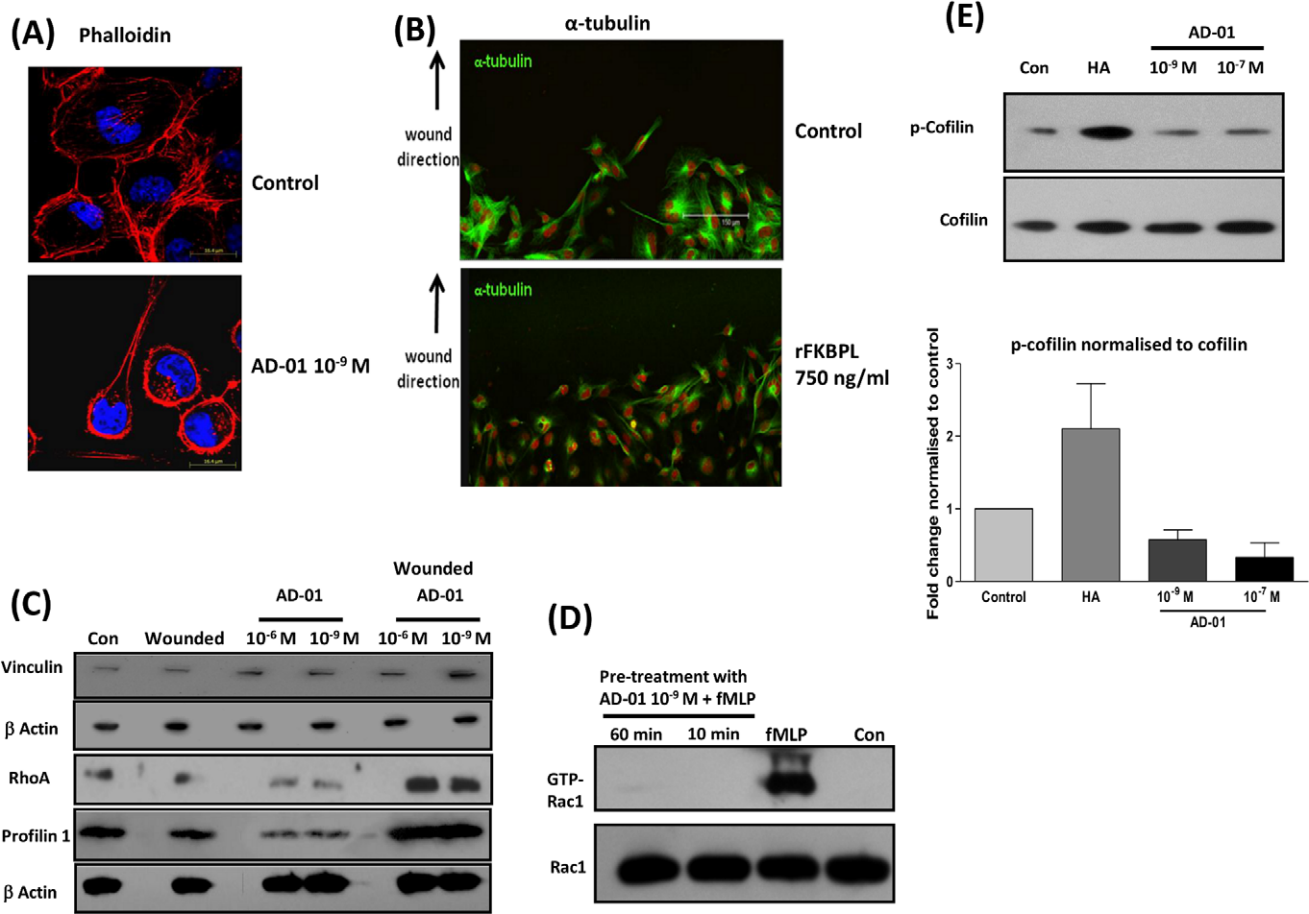
AD-01 and FKBPL mediate cytoskeletal changes **and AD-01 disrupts the RhoA-Rac1 dynamics.** (**A**) Confocal images (60x) representing the changes in phalloidin/F-actin dynamics in MDA-MB-231 upon wounding and treatment with AD-01 (10^−9 ^M) for 24 h; n = 3. Treated monolayers were fixed and stained with TRITC-phallodin (red) and DAPI (blue). Intense actin staining was accompanied by the loss in cell direction and communication. (**B**) Images (40x) representing disruption in tubulin distribution in HMEC-1 cell monolayers, wounded and treated with rFKBPL 750 ng/ml for 5 h. Fixed monolayers were stained for tubulin, followed by FITC conjugated secondary antibody (green) and nucleus with PI (red). (**C**) RhoA expression was increased after wounding and treatment with AD-01 for 24 h, resulting in a concomitant increase in the downstream actin binding proteins vinculin and profilin. Cells monolayers were treated with AD-01 (± wounding) for 24 h and total cell lysates were subjected to immuno blotting as indicated. (**D**) Treatment with AD-01 (10^−9 ^M) for 10/60 min inhibited fMLP induction (30 sec) of GTP-Rac-1 in HMEC-1 cells. Cell lysates of treated monolayers were subjected to Rac GTPase pull down assay; n = 3. (**E**) Representative western blots and quantitative densitometric analysis demonstrating an inhibition of cofilin phosphorylation after treatment with AD-01. HA treatment up-regulated cofilin phosphorylation maintaining its inactive state. HMEC-1 cell monolayers were treated with AD-01 for 3 h or HA for 10 min, and the extracted membrane fractions were subjected to immunoblotting with cofilin/p-cofilin; n = 3.

Although AD-01 up regulates CD44 expression, it appears to mediate an anti-migratory phenotype on CD44 positive cells, without effecting tumour or endothelial cell proliferation [11]. CD44 is involved in cell migration [25] and regulates the cytoskeleton by interacting with the ERM (ezrin/radixin/moesin) proteins. Therefore, to gain insight into the role of AD-01 and its regulation of cytoskeletal components required for cell migration we used confocal microscopy. Phalloidin staining of F-actin in MDA-MB-231 cells treated with AD-01 was significantly altered in comparison to the control cells (Fig. 5A). Untreated control cells wounded and deemed activated for migration showed fine and directional arrangement of F-actin enabling cell-cell contact. This organised staining pattern was completely lost 24 h post-treatment with AD-01, with cells showing intense cortical staining. HMEC-1 cells treated with rFKBPL showed a similar disruption in tubulin, possibly contributing to loss of cell direction and migration (Fig. 5B). This data is supported by evidence in several studies which demonstrated that cooperation between microtubules and the actin cytoskeleton is crucial in the control of cell shape, contraction, and motility [26]. We followed this up with further analysis of a number of proteins associated with Rho/Rac signalling pathway. Activation of the Rho GTPase family of proteins regulates the cytoskeletal structure of cells and their migratory functions, therefore RhoA, vinculin and profilin were chosen for further profiling in addition to Rac. An increase in RhoA, profilin and vinculin levels was indeed observed at 24 h following treatment and wounding of MDA-MB-231 cells with AD-01 (Fig. 5C). This effect was seen in cells wounded and deemed activated for directional migration; profilin and RhoA were not up-regulated in response to AD-01 in non-wounded cells which would not be activated for migration, indeed levels were lower than the control.

Since the temporal and spatial regulation of Rho and Rac-1 GTPase activation is essential for co-ordinated cell migration, we further examined the effect of AD-01 treatment on Rac-1 activity. Pre-treatment with AD-01 for 10 and 60 min completely abrogated the fMLP-mediated induction of Rac-1 activity, whilst AD-01 did not affect levels of total Rac-1 protein (Fig. 5D). This supported our previous findings demonstrating an AD-01 mediated inhibition of Rac-1 activation by serum as well as the CD44 ligand, HA [11]. Actin polymerisation is also regulated by the phospho-cycling of cofilin and treatment with AD-01 for 3 h inhibited the phosphorylation of cofilin therefore activating actin polymerisation (Fig. 5E). HA, however up-regulated cofilin phosphorylation maintaining its inactive state. Altogether the data highlights the role of FKBPL and AD-01 on the CD44-cytoskeletal pathway leading to inhibition of cell migration.

## Discussion

FKBPL is a non-canonical member of the FK506 immunophilin family and has several distinct and important cellular roles in regulating the process of malignancy [12]–[18]. Using overexpressed or rFKBPL, we have recently shown that FKBPL is an anti-angiogenic protein and its activity resides in a 24 amino acid region [11]. In this study, siRNA knockdown reversed the anti-angiogenic effects of the endogenous protein. Although FKBPL lacks the conventional signal peptide domain required for secretion via Golgi-ER trafficking [27], we have previously reported its secretion [11]. Further evidence of its extracellular function comes from the observation that migrating endothelial cells when exposed to anti-AD-01 antibody raised against FKBPL’s active domain demonstrate accelerated migration, possibly due to abrogation of the anti-migratory activity of secreted FKBPL by the blocking antibody. Intracellular compartmentalisation of FKBPL in the cytosol, membrane and nuclear fractions coupled with its punctate staining, indicative of vesicular patterning, would support its various cellular roles in each of these compartments in addition to its already well characterised intracellular roles in nuclear steroid hormone receptor complexes [15], [17].

*In vivo*, FKBPL overexpressing tumour xenografts develop a compromised tumour vasculature consistent with FKBPL’s role as a secreted anti-angiogenic protein (Fig. 1E). Although secretion of FKBPL has not been observed by the parental MDA-MB-231 cells, FKBPL overexpressing MDA-MB-231 cells demonstrated FKBPL secretion *in* *vitro* [11]; this secretion, if occurring *in* *vivo* would support the FKBPL-mediated anti-angiogenic effects on the murine tumour endothelium. We have reported similar effects with the exogenous FKBPL-based therapeutic peptide, AD-01, in prostate xenografts in mice following systemic administration for 14 days [11]. This data further supports a role for FKBPL as a secreted protein with anti-angiogenic activity, where high levels also correlate with an increase in overall survival and metastasis-free survival observed in clinical breast cancer data sets [17]. In our previous study [11] we have reported that FKBPL’s anti-migratory activity is restricted to cell lines expressing CD44. Here, we provide further evidence to suggest that both endogenous FKBPL and its peptide derivative, AD-01, interact with CD44 resulting in cytoskeletal disruption. In order to further interrogate this interaction, surface plasmon resonance was employed. However, attempts to establish an assay system between AD-01, rFKBPL, and CD44’s natural ligand, HA, using rCD44 immobilised on a Sensor Chip CM5 in a cell free system were unsuccessful (Fig. S1). Since the FKBP family of proteins are involved in protein-protein interactions [28], the possibility that the FKBPL/AD-01 and CD44 interaction requires other factors/proteins cannot be ruled out. To address this, an indirect assay was constructed to identify AD-01 interactions with CD44 derived directly from fresh cell extracts. In MDA-MB-231 cells, binding of immunopurified CD44 with AD-01 was detected and this interaction was more significant at lower concentrations of AD-01 as the interaction with anti-AD-01 antibody was competed out at the higher AD-01 concentrations. Knockdown studies using crude cell extracts of MDA-MB-231 provided further evidence that AD-01 has a requirement for CD44 to mediate its anti-migratory activity.

We then focused on the activation/inactivation of candidate proteins that are known to be involved in the Rho/Rac/CD44 pathway. CD44 has a well-established role in breast cancer invasion/metastasis [29] and angiogenesis [30] and is subject to extensive splicing and post-translational modification [31]. There are a number of reports correlating the expression of CD44 variants in metastatic spread [32], [33], and their therapeutic targeting for tumour control [34], [35]. The standard isoform, CD44s, has also been attributed with tumour suppressor functions in carcinomas of the breast [36]–[38], prostate [39], [40] and colon [41]. The pro- and anti-tumour functions of CD44 are not merely determined by its presence but are also governed by other factors; ligand binding, isoforms, structure, post-translational modifications, complexing of its cytoplasmic tail with other proteins, subcellular locations and downstream signalling pathways [42], [43]. Upon binding to cell surface CD44, AD-01 as well as endogenous FKBPL, trigger downstream signalling events and up-regulate CD44 expression at the protein and mRNA level. The AD-01-mediated up-regulation of FKBPL would also possibly result in the modulation of CD44 levels, as we demonstrate that siRNA-mediated knockdown or stable overexpression of FKBPL correlate with CD44 levels (Fig. 4); it is highly likely that these two interacting proteins therefore regulate each other’s activity and mediate downstream effects on cell migration, leading to an upregulation of signalling through the focal adhesion pathway and an increase in RhoA and its effector proteins (Fig. 5). Expression of actin-binding proteins, profilin and vinculin, is associated with decreased invasion, and cell migration [44], [45], supporting our own data where we demonstrate an AD-01-mediated increase in profilin and vinculin correlating with decrease cell motility. A balance of RhoA and Rac1/Cdc42 GTPase activity is required during cell migration. Whilst Rac1/Cdc42 activity mediates leading edge migration, RhoA acts at the trailing edge and there is an inverse relation between the two GTPases [46], [47]. AD-01 treatment appears to disrupt the cytoskeleton and affects the balance of Rho-Rac activity leading to a loss of cell rolling dynamics and cell-cell communication eventually inhibiting cell migration. In support of our data, constitutive activation of RhoA, mediated similar effects on stress fibre formation and cytoskeletal disruption leading to impaired motility in MDA-MB-231 cells [48]. Whilst there appeared to be a downregulation of RhoA and profilin following addition of AD-01 in non-wounded cells (Fig. 5C), this would be irrelevant to a developing vasculature where endothelial cells would be activated to migrate, although further investigation would be required to address this effect.

Additional evidence towards the disruption of actin comes from the AD-01 mediated effect on the activation of cofilin which is also reported to result in inhibition of collective cell migration [49] due to inhibition of Rac-1 activation. Whilst it is clear that FKBPL and AD-01 bind and regulate CD44, the precise mechanisms leading to their downstream effects on the cytoskeleton are not clear, nor whether the changes we see in cell signalling are the cause or consequence of cytoskeletal disruption. It is likely that FKBPL and its peptide derivatives bind to a CD44 complex at the cell surface are internalised via CD44 and mediate direct effects on the cytoskeleton leading to the loss of functional cell migration and consequent inhibition of Rac-1 activity. Interaction of FKBPL with the cytoskeleton via complexing with microtubule binding protein dynamitin has been reported earlier [15]. Inhibition of Rac-1 possibly leads to up-regulation of RhoA and downstream effector molecules resulting in increased focal adhesion and cortical staining. Since normal migratory functions of the cell are compromised, signalling for further CD44 synthesis could be initiated, consistent with the up-regulation demonstrated upon AD-01 treatment. Further investigation into the effect of AD-01 on CD44 internalisation and its interactions with ERM, ankyrin and RHAMM, will provide a better understanding of this mechanism and further insight into the role of CD44 in modulating the cytoskeleton and tumourigenesis.

### Conclusions

We have identified that FKBPL and its peptide derivative, AD-01, can interact with CD44, regulate CD44 signalling and expression levels and interfere with Rho/Rac signalling in wound-activated cells leading to reduced cell migration. Given limited success from VEGF-targeting approaches and positive correlations between CD44 expression and enhanced angiogenic properties of cancer cells [50], the FKBPL peptide may prove advantageous in the field of novel anti-angiogenic agents currently in clinical use.

## Supporting Information

Figure S1 Sensograms demonstrating lack of binding of **(A)** CD44 ligand, HA (0.1 µg/ml); anti-CD44 antibody was used as a positive control or **(B)** AD-01, Scr AD-01, rFKBPL to rCD44 immobilised on CM5 chip. The data indicate that the process of immobilisation mediated a conformational change in the active site of CD44, preventing binding, even to its natural ligand HA, although immunogenicity is retained as demonstrated
(TIFF)
