# Supplementary figures and images for: The Anti-Migratory Effects of FKBPL and Its Peptide Derivative, AD-01: Regulation of CD44 and the Cytoskeletal Pathway
Source: PLoS One. 2013 Feb 15;8(2):e55075. doi: 10.1371/journal.pone.0055075 (PMC3574160; doi:10.1371/journal.pone.0055075)

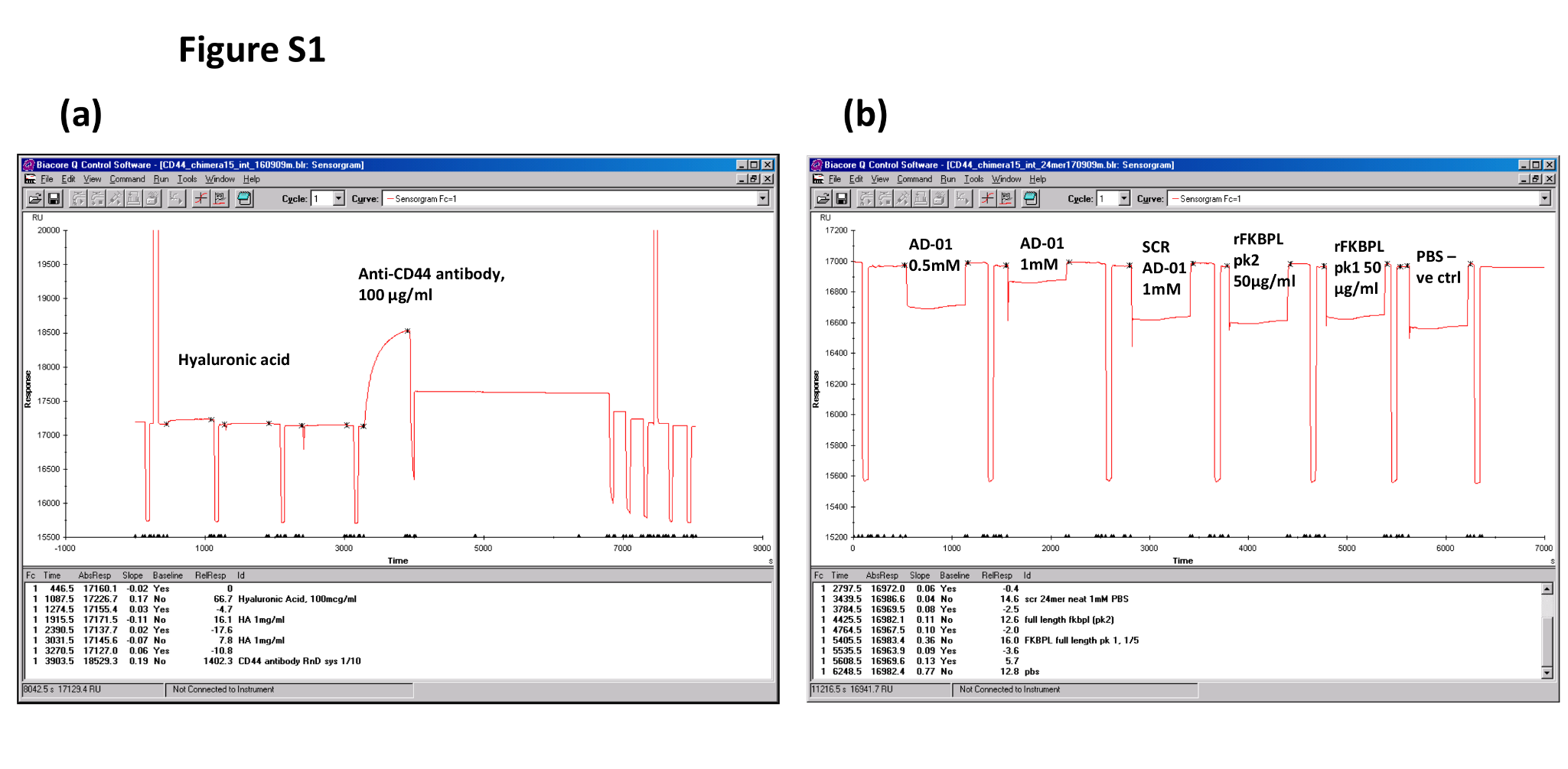

Supplement: Figure S1 — Sensograms demonstrating lack of binding of (A) CD44 ligand, HA (0.1 µg/ml); anti-CD44 antibody was used as a positive control or (B) AD-01, Scr AD-01, rFKBPL to rCD44 immobilised on CM5 chip. The data indicate that the process of immobilisation mediated a conformational change in the active site of CD44, preventing binding, even to its natural ligand HA, although immunogenicity is retained as demonstrated (TIFF) [file pone.0055075.s001.tiff]
